# 9‐Deoxymuzigadial, a Sesquiterpene Isolated From *Drimys brasiliensis* (Winteraceae), Displays Reduced Cytotoxicity In Vitro and Modulates Leukocyte Activity and Fibrogenesis In Vivo

**DOI:** 10.1002/cbdv.202503329

**Published:** 2026-01-08

**Authors:** Bruno Antonio Ferreira, Isabella Silva Cassimiro, Francyelle Borges Rosa de Moura, Tais de Campos Lima, Danielle Reis Napolitano, Eric Umehara, João Henrique Ghilardi Lago, Fernanda de Assis Araújo

**Affiliations:** ^1^ Center for Natural and Human Sciences Federal University of the ABC Santo Andre Brazil; ^2^ Institute of Biomedical Sciences Federal University of Uberlândia Uberlândia Brazil; ^3^ Institute of Biotechnology Federal University of Catalão Catalão Brazil; ^4^ Federal University of São João Del‐Rei Divinópolis Brazil

**Keywords:** collagen, drimane sesquiterpene, inflammation, natural products, sponge implants

## Abstract

The aims of this study were to investigate in vitro cytotoxic potential and the effects of daily administration during the inflammatory response induced by sponge implants in mice of the sesquiterpene 9‐deoxymuzigadial (9‐DOM), isolated from *Drimys brasiliensis* (Winteraceae). Initially, 9‐DOM showed no cytotoxic activity in RAW264.7 macrophages. In implants treated with 0.1 µg of 9‐DOM, a reduction in macrophage activity and in the average number of mast cells were observed. In addition, a pro‐fibrogenic effect was observed, with an increase in the synthesis and deposition of collagen, particularly thinner collagen fibers. None of the doses evaluated were able to alter the parameters associated with angiogenesis assessed. Although initial, the data demonstrates the low toxicity of 9‐DOM and its possible therapeutic application in situations where exacerbated inflammation and low collagen synthesis and deposition can compromise tissue repair.

## Graphical abstract

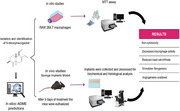

## 1 Introduction

Previous studies have linked the anti‐inflammatory effects of different sesquiterpenes to the inhibition of proinflammatory signaling pathways, the synthesis of proinflammatory mediators, and/or the recruitment and activity of inflammatory cells [1]. Although inflammation is fundamentally a protective response aimed at eliminating harmful agents, cellular debris, and ultimately initiating subsequent tissue repair, the chronicity of the inflammatory process can create a microenvironment that facilitates the progression of certain pathologies [2]. While several drugs target this process, prolonged use of many available anti‐inflammatory drugs can lead to undesirable side effects. In this context, the search for new anti‐inflammatory drugs derived from natural products has garnered significant scientific and medical–pharmaceutical interest, as they represent potential prototypes for the development of new drugs with low toxicity [3, 4].

*Drimys brasiliensis* Miers. (Winteraceae) is a native species of the Brazilian flora, found in the Cerrado and Atlantic Forest biomes [5]. Its bark and leaves are traditionally used in folk medicine for their anti‐inflammatory and antinociceptive properties. Furthermore, this plant has been commonly employed in the treatment of ulcers, cancer, respiratory issues and parasitic infections [6, 7, 8]. Phytochemically, *D. brasiliensis* produces different sesquiterpenes, especially drimane derivatives such as polygodial and 9‐deoxymuzidial (9‐DOM) [9]. 9‐DOM has been reported in other plant species, including the stem bark and leaves of *Canella winterana* [10, 11], the leaves and fruits of several *Pseudowintera* species (*P. colorata*, *P. axillaris*, and *P. insperata*) [12, 13], and the stem bark of *Warburgia ugandensis* [14]. To date, research has primarily focused on its antiparasitic activity, with studies evaluating its efficacy against protozoan pathogens such as *Trypanosoma cruzi* [15], *Schistosoma mansoni* [5], and *Plasmodium falciparum* [16], as well as its insecticidal properties [12]. Notably, no studies have yet explored the potential effects of 9‐DOM on biological processes such as inflammation or fibrogenesis. Therefore, our objective was to evaluate the in vitro cytotoxic potential of this compound, as well as to investigate the effects of its daily in vivo administration in a chronic inflammation model induced by polyester–polyurethane sponge implants. This model enables the assessment of inflammatory cell infiltration and key processes such as angiogenesis and fibrogenesis [17]. In addition to the biological evaluations, the study also aimed to perform an in silico ADME (absorption, distribution, metabolism and excretion) analysis of 9‐DOM to predict its in silico pharmacokinetic behavior and support its potential as a therapeutic candidate. The results obtained indicate that 9‐DOM exhibits low in vitro cytotoxicity and that its intra‐implant administration, at least at the lowest dose tested, reduces the activity and number of certain inflammatory cells while promoting the fibrogenesis process.

## 2 Results and Discussion

### 2.1 Chemical Characterization of 9‐DOM

**FIGURE 1:**
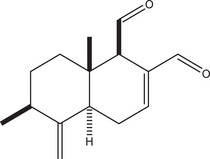
Structure of sesquiterpene 9‐deoxymuzigadial (9‐DOM).

^1^H and ^13^C NMR as well as MS data of 9‐deoxymuzigadial (9‐DOM; Figure 1), obtained in 99% of purity as indicated by HPLC, were compared with those reported in the literature [10].

### 2.2 In Silico Evaluation of 9‐DOM

**FIGURE 2:**
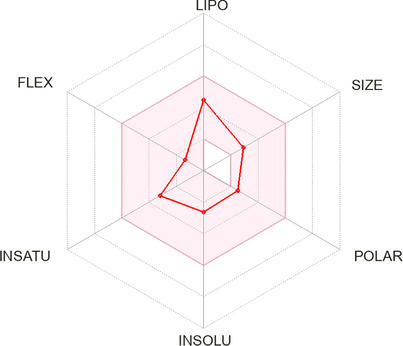
Bioavailability radar of 9‐deoxymuzigadial (9‐DOM) generated using SwissADME. The radar graph provides a visual representation of six physicochemical properties relevant to oral bioavailability.

The in silico analysis of 9‐DOM was carried out using the free SwissADME web tool (http://www.swissadme.ch/). The similarities between the tested compound and known drugs are clearly illustrated by the Bioavailability Radar (Figure 2), which evaluates six key physicochemical properties: lipophilicity (LIPO), molecular size (SIZE), polarity (POLAR), solubility (INSOLU), saturation (INSATU) and flexibility (FLEX). The pink area on the radar delineates the optimal range for each parameter, within which a compound is considered to exhibit drug‐like characteristics. Table 1 provides a detailed overview of the physicochemical properties of 9‐DOM, including molecular descriptors relevant to drug‐likeness evaluation, such as compliance with Lipinski's rule of five [18, 19]. Table 2 presents the predicted pharmacokinetic parameters of the compound.

**TABLE 1:** Molecular properties and in silico drug‐likeness of 9‐deoxymuzigadial (9‐DOM).

Parameters	9‐deoxymuzigadial
Molecular weight (Da)	232.32
TPSA (Å)	34.14
log *P*_o/w_	2.17
log *S*	−2.63
Lipinski	Yes; 0 violation
Ghose	Yes
Veber	Yes
Egan	Yes
Muegge	Yes
Bioavailability score	0.55
PAINS	0 alert

**TABLE 2:** In silico pharmacokinetic parameters of 9‐deoxymuzigadial (9‐DOM).

Parameters	9‐deoxymuzigadial
GI absorption	High
BBB permeant	Yes
P‐gp substrate	No
CYP1A2 inhibitor	No
CYP2C19 inhibitor	No
CYP2C9 inhibitor	No
CYP2D6 inhibitor	No
CYP3A4 inhibitor	No
log *K*_p_ (skin permeation)	−6.05 cm/s

Lipophilicity (log *P*_o/w_) and solubility (log *S*) are critical physicochemical properties of a drug, as they are, respectively, associated with its capacity to permeate cellular membranes and to ensure the availability of an adequate amount of the active compound in a minimal dosage form [20]. A molecule's drug‐likeness potential is assessed using five distinct rule‐based filters, each defined by a specific set of physicochemical and structural properties [18]. These values suggest a high absorption potential and favorable oral bioavailability, supporting the classification of 9‐deozymuzigadial as a promising lead‐like compound. Moreover, the absence of pan‐assay interference compounds (PAINS) alerts further reinforce its potential as a viable drug candidate.

The predicted high gastrointestinal absorption of 9‐DOM indicates favorable pharmacokinetic properties for oral administration. The moderate to low skin permeability (log *K*_p_) suggests limited transdermal absorption, which may reduce systemic exposure via this route. The absence of interaction with P‐glycoprotein (P‐gp) may further enhance its oral bioavailability, as efflux by P‐gp often limits the absorption of xenobiotics [21, 22]. The predicted ability of 9‐DOM to cross the blood–brain barrier (BBB), combined with its lack of interaction with P‐gp, raises concerns about unintended central nervous system (CNS) exposure. Such characteristics may lead to off‐target CNS effects, particularly in therapeutic contexts where the CNS is not the primary site of action [21]. Further pharmacological and toxicological studies are necessary to elucidate its effects on the CNS and ensure its safety profile.

Cytochrome P450 (CYP) enzymes play a central role in the Phase I metabolism of xenobiotics. These enzymes, particularly isoforms such as CYP3A4, CYP2D6, CYP2C9, and CYP2C19, catalyze oxidative reactions that increase the polarity of drug molecules, facilitating their subsequent conjugation and excretion [23]. In drug development, the absence of inhibitory activity against major CYP isoforms is considered a favorable characteristic. Compounds that do not inhibit these enzymes are less likely to cause drug–drug interactions, which enhances their safety profile. Moreover, such compounds tend to exhibit more predictable pharmacokinetic behavior, contributing to consistent therapeutic outcomes across diverse patient populations [24].

### 2.3 Evaluation of Toxicity In Vitro of 9‐DOM

**FIGURE 3:**
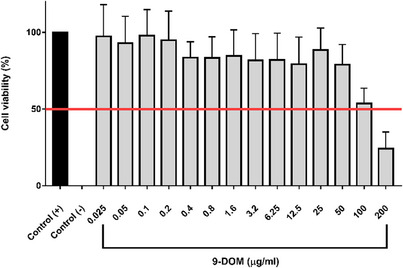
The cytotoxic effects of sesquiterpene 9‐deoxymuzigadial (9‐DOM) on RAW264.7 murine macrophages cell line. Positive control cells were treated with medium containing 5% FBS alone, while negative control cells were treated with 10% DMSO. Data are presented as mean ± SEM of three separate experiments.

Cytotoxicity potential of 9‐DOM was accessed using RAW264.7 macrophages (Figure 3). The cytotoxicity assay demonstrated that 9‐DOM exhibited low toxicity against the observed cell line, with an inhibitory concentration (IC_50_) of 102.3 µg/mL. This low toxicity was also observed in VERO cells in vitro [5].

### 2.4 Daily Administration of 9‐DOM Attenuates Macrophage Activity and Mast Cell Content Near the Sponge Implants

The implantation of sponge matrices constitutes a well‐established experimental model for the quantitative assessment of inflammatory, angiogenic, and fibrogenic responses, which are commonly observed in pathophysiological contexts such as chronic inflammation and wound healing. These matrices function as three‐dimensional scaffolds that support the formation of fibrovascular tissue, characterized by the infiltration of inflammatory cells, neovessel formation, and the recruitment of fibroblasts and other stromal components [25, 26]. A notable advantage of this model is its capacity to allow controlled modulation of the local microenvironment, which facilitates the evaluation of how different bioactive compounds influence these key biological processes, thereby providing insights into their mechanisms of action in tissue remodeling [26, 27].

**FIGURE 4:**
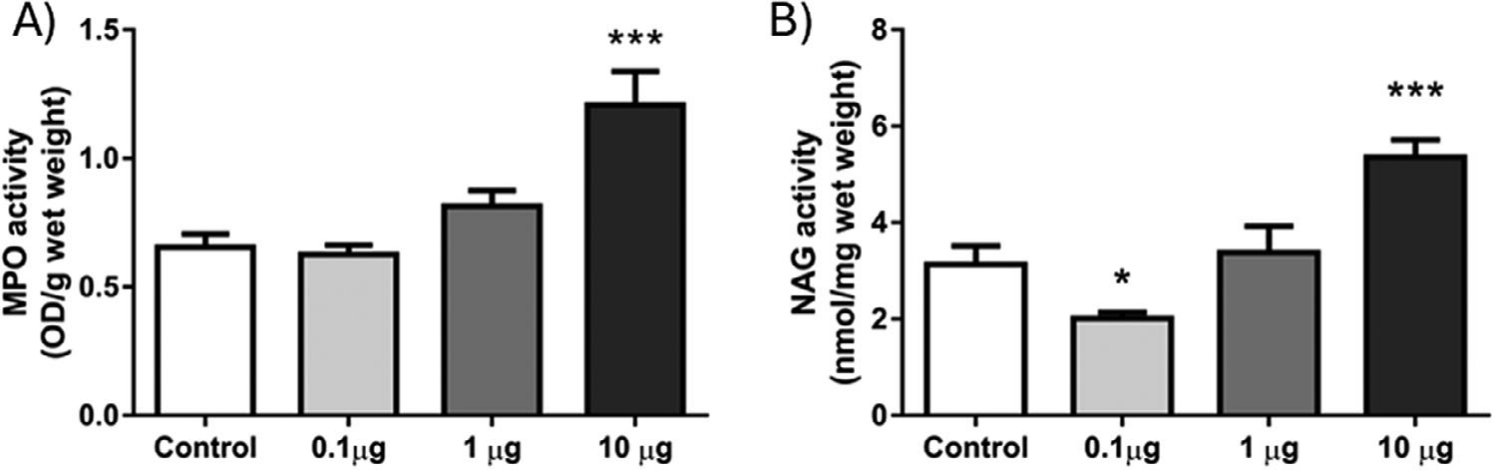
Effects of daily and intra‐implant administration of 9‐deoxymuzigadial (9‐DOM) on neutrophil and macrophage activity. (A) Myeloperoxidase activity (MPO) and (B) *N*‐acetyl‐β‐d‐glycosaminidase activity (NAG). *n* = 8–10 animals. *p < 0.05***p < 0.001. ANOVA, Bonferroni posttest.

In our study, neutrophil and macrophage infiltration in sponge discs was assessed using the activity of the enzymes myeloperoxidase (MPO) and *N*‐acetyl‐β‐d‐glucosaminidase (NAG), respectively (Figure 4A,B). Daily intra‐implant administration of 9‐DOM at its lowest dose was able to attenuate NAG activity when compared to the control group. However, at the highest dose, administration of 9‐DOM promoted greater activation of both cell types, with mean values for MPO and NAG significantly increased compared to the control group.

Neutrophils are generated in the bone marrow through granulopoiesis and serve as the first line of defense during acute inflammation. They represent the most abundant leukocyte population in human peripheral blood [28]. Upon sensing chemotactic gradients of cytokines and chemokines, neutrophils rapidly migrate to sites of tissue injury or infection. These cells express a diverse repertoire of pattern recognition receptors, enabling the detection of pathogen‐associated molecular patterns (PAMPs) and damage‐associated molecular patterns (DAMPs). Although short‐lived, neutrophils possess a potent microbicidal arsenal. However, their sustained activation within tissues can contribute to collateral damage and exacerbate inflammation [29]. Macrophages are recruited to inflamed sites at later stages and exhibit remarkable phenotypic plasticity. During the inflammatory response, they participate in multiple processes, ranging from the clearance of harmful stimuli to the resolution of inflammation and tissue repair [30]. Upon infiltration into inflamed tissues, macrophages typically acquire a proinflammatory phenotype, which may persist under chronic pathological conditions, thereby contributing to disease progression. Otherwise, the transition to an anti‐inflammatory macrophage phenotype involves a cascade of events, including the presence of specific soluble mediators in the microenvironment, the efferocytosis of apoptotic neutrophils, and oxidative stress itself [31, 32]. Therefore, the discrepancy observed between the groups treated with the lowest and highest doses of 9‐DOM, with respect to macrophage proinflammatory activity (NAG activity), may be associated with the persistence of the neutrophilic infiltrate at the highest dose (MPO activity), whose mechanisms of action also encompass the generation of reactive oxygen species (ROS).

**FIGURE 5:**
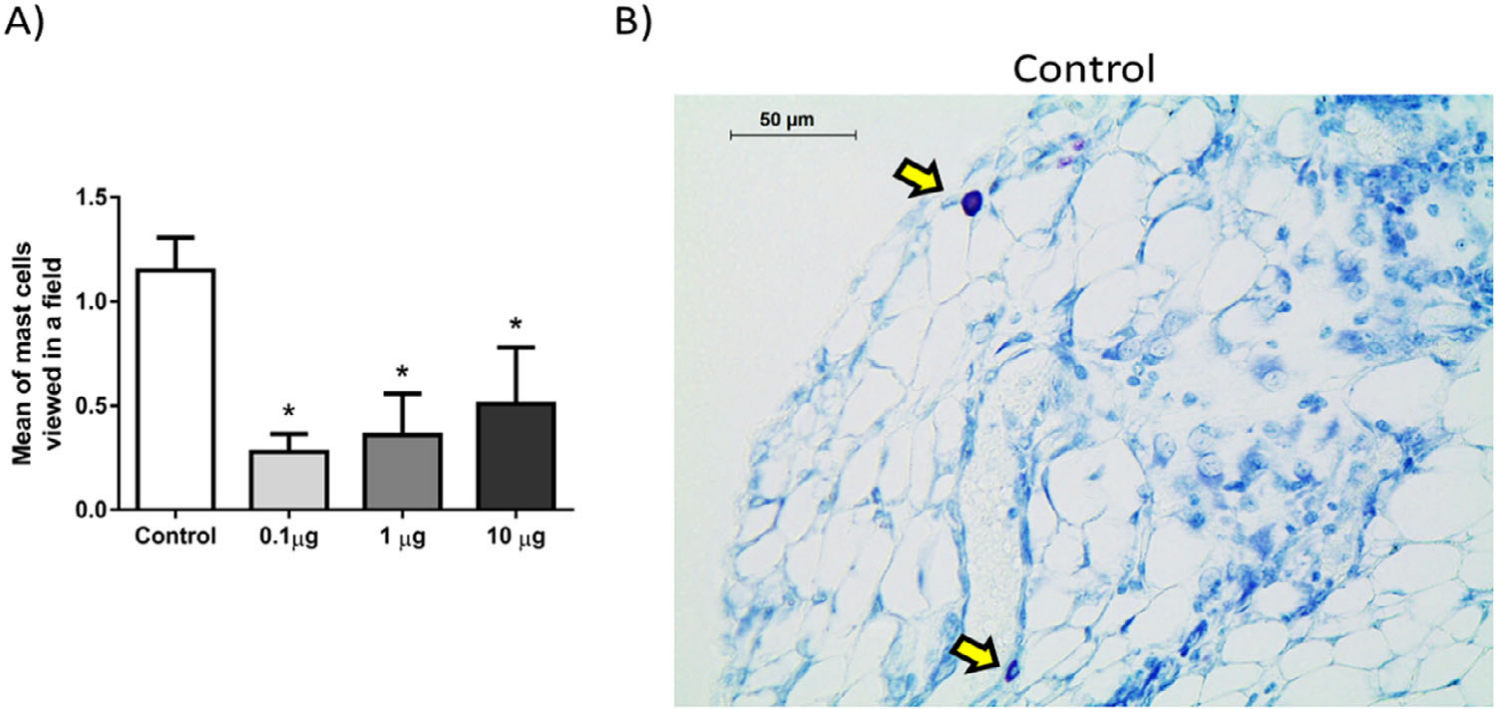
Treatment with the sesquiterpene 9‐deoxymuzigadial (9‐DOM) decreased the number of mast cells present near the sponge implant. (A) Graphical representation of the average number of mast cells seen in the fields observed. (B) Photomicrograph of the sections (5 µm) stained with toluidine blue (Scale bar = 50 µm). The mast cells are purple thanks to the metachromasia of their granules. *n* = 6 animals. *p < 0.05. ANOVA, Bonferroni posttest.

9‐DOM also reduced the presence of mast cells near the sponge implants (Figure 5). Mast cells are involved in responses such as allergies and inflammation. The process of mast cell degranulation, with the release of pre‐stored substances from their granules, is one of the organism's first responses to identifying a harmful insult. In addition, mast cells are a source of proinflammatory mediators, such as cytokines and growth factors, which help recruit and activate leukocytes at the inflammatory site [33, 34].

According to our results, 9‐DOM modulates the activity and accumulation of inflammatory cells in the tissue surrounding the implants in a dose‐dependent manner, exerting either proinflammatory or anti‐inflammatory effects. Notably, treatment with the lowest concentration of 9‐DOM produced effects comparable to those observed with polygodial, a structurally analog sesquiterpene. Similar to the findings presented here, polygodial administration has been reported to reduce macrophage activity (as indicated by NAG) and mast cell density around sponge implants, without affecting neutrophil activity (MPO). This study also reported a reduction in the levels of the proinflammatory chemokines CXCL1 and CCL2 in the treated groups [35]. Previous studies have shown that polygodial can attenuate the expression of the transcription factor NF‐κB. Moreover, treatment with polygodial reduces the expression and phosphorylation of the NF‐κB inhibitor protein IκB, a key step required for NF‐κB nuclear translocation and the consequent transcription of proinflammatory genes [36]. Additional evidence has revealed anti‐inflammatory effects, comparable to those induced by dexamethasone, in polygodial‐treated pancreatic β cells. These effects were mainly associated with inhibition of the MAPK/ERK1/2 axis, whose activation is implicated in autoimmune and chronic inflammatory disorders [37]. Considering the structural similarity between these molecules, it is plausible that the effects elicited by 9‐DOM involve similar mechanisms. However, further studies are necessary to confirm this assumption.

The anti‐inflammatory properties of chemically related sesquiterpenes are associated with several mechanisms, including the inhibition of inflammatory cell recruitment through downregulation of cell adhesion molecules (e.g., ICAM and VCAM) [38], suppression of proinflammatory mediators, such as cytokines, chemokines and products of arachidonic acid metabolism (e.g., 12(*S*)‐HETE and LTB_4_) [39, 40].

### 2.5 Administration of 9‐DOM Did Not Alter the Formation of New Blood Vessels

**FIGURE 6:**
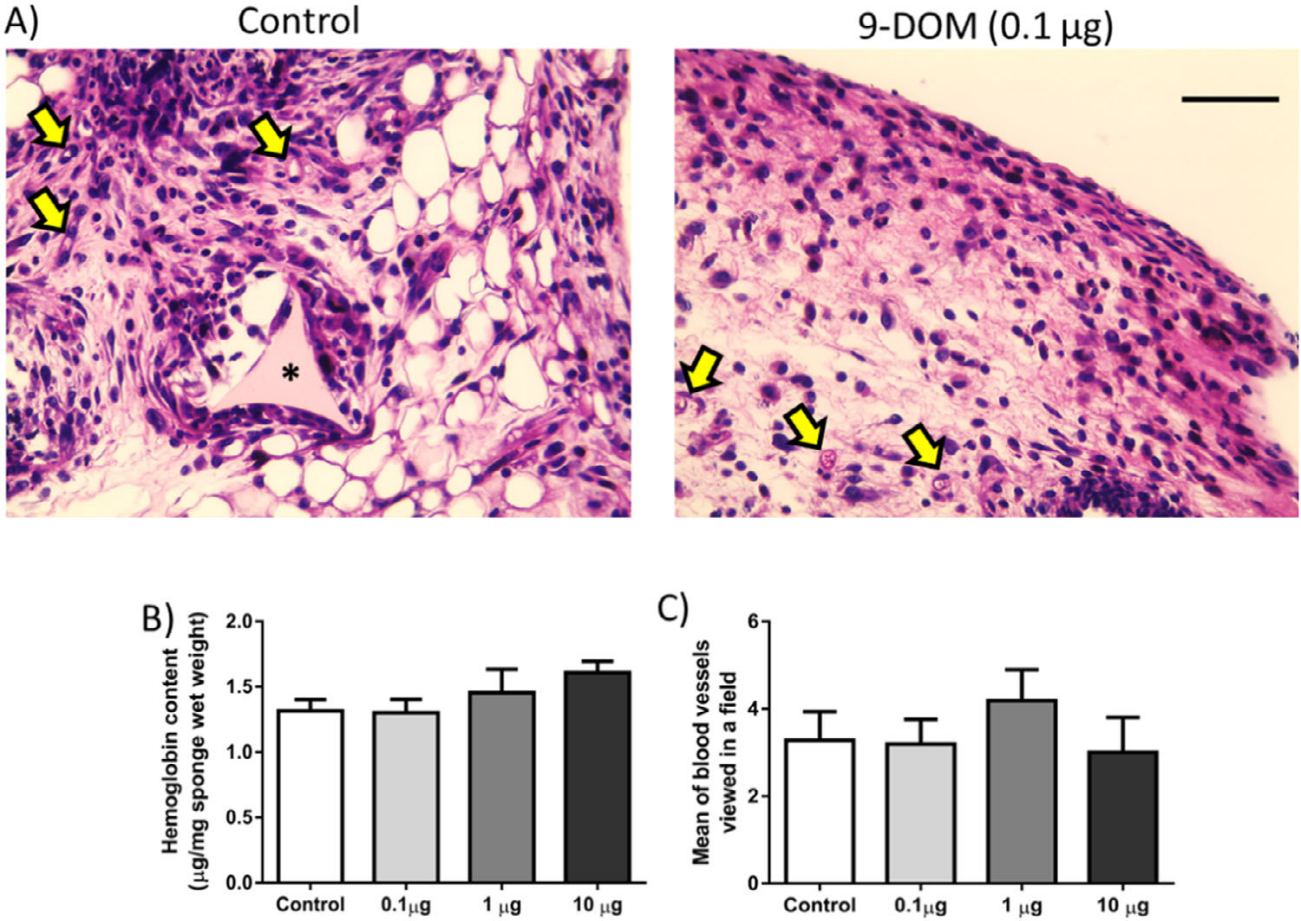
Effects of treatment with 9‐deoxymuzigadial (9‐DOM) on the angiogenesis process. (A) Photomicrograph of the implants stained with hematoxylin and eosin (scale bar = 50 µm). The pores of the implants are filled by the infiltration of inflammatory cells, blood vessel, fibroblasts, and the deposition of ECM constituents. (B) Hemoglobin content near the implants (*n* = 8–10 animals). (C) Graphical representation of the average number of blood vessels observed in the histological sections (*n* = 6 animals). ANOVA, Bonferroni posttest.

Angiogenesis, the process of new blood vessel formation, although independent, can be influenced by mediators from the inflammatory site. On the other hand, the formation and maintenance of an excessive vascular network also contribute to the chronicity of the inflammatory response, as it supplies nutrients, oxygen, and leukocytes to this microenvironment [41, 42]. In this study, the formation of new blood vessels was assessed by counting blood vessels in histological sections stained with hematoxylin and eosin (H&E), as well as by quantifying hemoglobin content (Figure 6). Although several sesquiterpenes may affect inflammatory angiogenesis by altering the synthesis of proangiogenic mediators [43], the activity of matrix metalloproteinases (MMPs) [44] or inhibiting the migration and proliferation of endothelial cells, in the present study [45], the administration of 9‐DOM did not alter the formation of new blood vessels or the hemoglobin content in the fibrovascular tissue induced by the sponge implants (Figure 5). This inability to alter the angiogenic parameters evaluated was also observed during the administration of the chemically related sesquiterpene polygodial [35].

### 2.6 Deposition and Organization of the Extracellular Matrix Differs According to the Doses Administered of 9‐DOM

**FIGURE 7:**
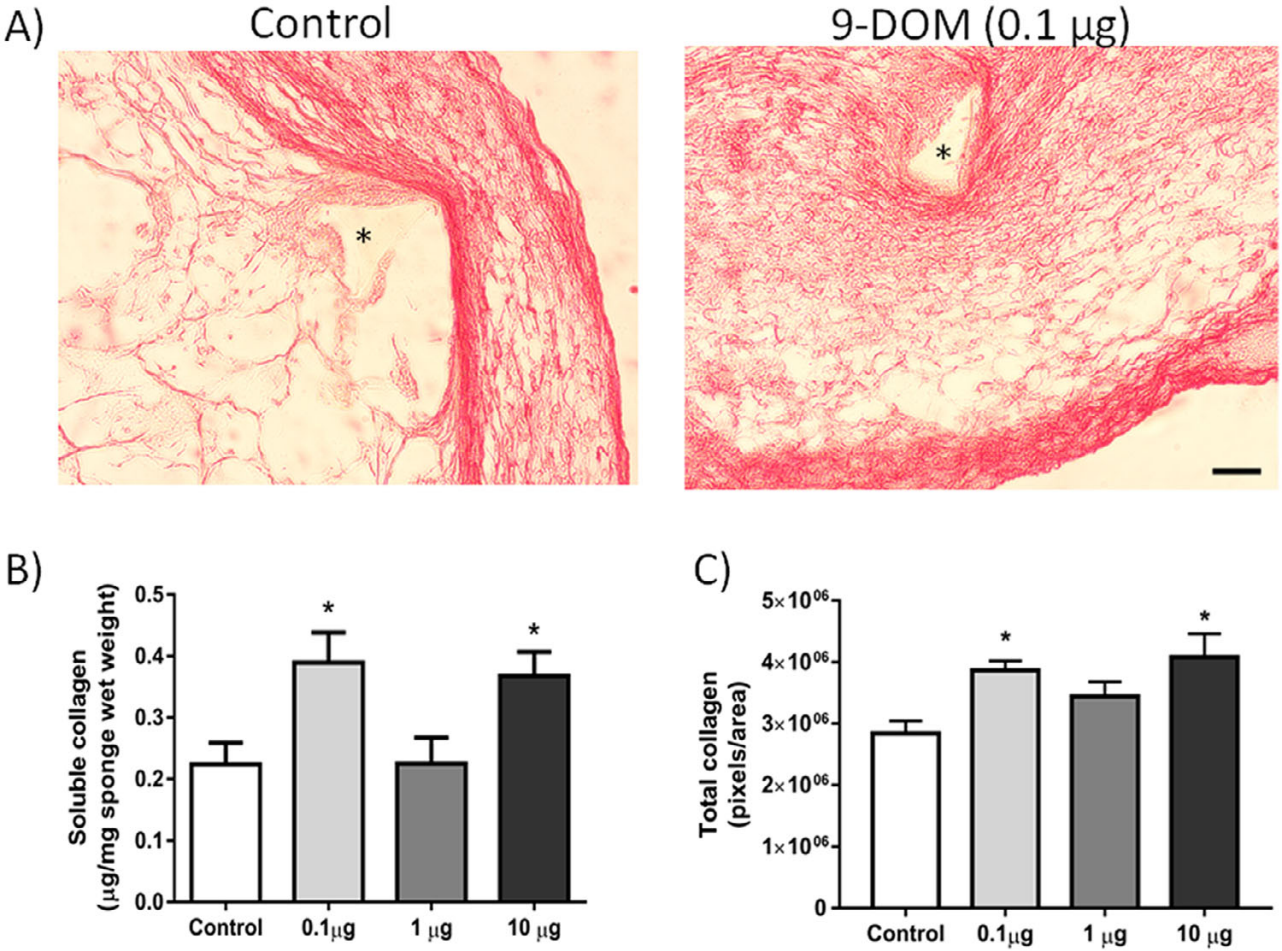
Effects of 9‐deoxymuzigadial (9‐DOM) administration on fibrogenesis. (A) Photomicrographs of histological sections stained with picrosirius red (Scale bar = 50 µm). (B) Graphical representation of soluble collagen content (*n* = 8–10 animals). (C) Total collagen deposited near the implants, quantified from histological sections stained with picrosirius red (*n* = 6 animals). *p < 0.05 versus control group. ANOVA, posttest Bonferroni.

Fibrogenesis refers to the synthesis of collagen, an important structural protein that helps maintain the properties of the extracellular matrix (ECM), in association with other components such as glycosaminoglycans, proteoglycans, and adhesive glycoproteins [46]. Fibrillar collagens, particularly Types I and III, are the main constituents of the ECM, forming bundles that can be observed under optical microscopy [47]. In the implants treated with 0.1 and 10 µg of 9‐DOM, an increase in collagen synthesis and deposition compared to the control group was observed (Figure 7).

**FIGURE 8:**
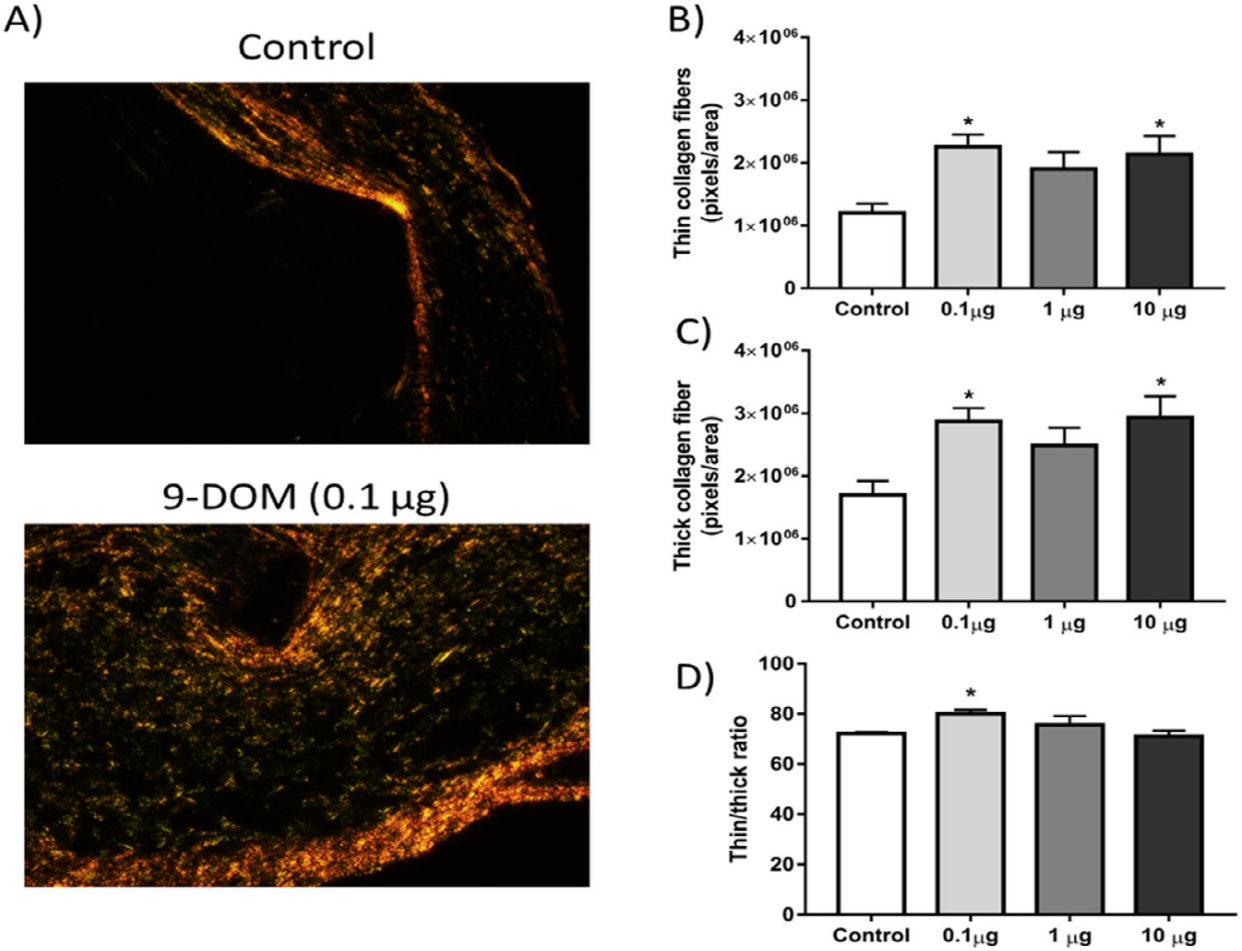
Treatment with 9‐deoxymuzigadial (9‐DOM) promoted a change in the organization of the extracellular matrix. Photomicrographs of the implants after 9 days of treatment with the sesquiterpene. The sections stained with picrosirius red were observed using a polarized light filter. In green, normally corresponding to Type III collagen fibers, we can see thinner fibers. In orange/red, corresponding to Type I collagen fibers, we see thicker fibers. Graphical representations of (A) the density of thin collagen fibers, (B) thick collagen fibers, and (C) the ratio between thin and thick fibers. Results represent the mean ± SEM (*n* = 6 animals per group). *p < 0.05 versus control group. ANOVA, posttest Bonferroni.

However, in animals treated with 0.1 µg of 9‐DOM, when evaluating the organization of ECM constituents, a higher proportion of thinner collagen fibers was observed, which are generally associated with Type III collagen (Figure 8). The higher proportion of this collagen type may be linked to the attenuation of the inflammatory response, which was also observed after administration of 9‐DOM at 0.1 µg.

Using this same model, a similar effect after daily administration of the sesquiterpene α‐zingiberene, which was capable of inhibiting the activity of MMP‐2 and ‐9 enzymes, important for ECM remodeling, was previously reported [44]. However, when the obtained results of 9‐DOM were compared to those previously reported to the chemically related sesquiterpene polygodial, showed opposite effects [35]. These effects indicate that the structural differences between these molecules play an important role in the observed biological effects.

Finally, some studies highlight the consequences of ECM composition and organization in the repair process. For example, in fetal skin wound healing, the injured tissue regenerates without forming scars. The higher proportion of Type III collagen compared to adult skin is one of the factors contributing to this difference [48, 49].

## 3 Conclusions

In summary, the data presented demonstrate the anti‐inflammatory and pro‐fibrogenic potential of 9‐DOM, a drimane sesquiterpene isolated from Brazilian plant *D. brasiliensis*, when administered at reduced concentrations. As observed 9‐DOM was shown to reduce part of the inflammatory infiltrate near the sponge implants, while stimulating collagen synthesis and deposition, particularly of thinner collagen fibers (Type III collagen). In addition, 9‐DOM demonstrated low cytotoxicity in in vitro assays and exhibited favorable properties in in silico ADME analysis. Although these findings are promising and suggest the therapeutic potential of 9‐DOM, further studies are required to elucidate the mechanisms underlying the effects of this sesquiterpene. Comprehensive pharmacokinetic and toxicity evaluations are also essential to confirm its safety profile. Taken together, the results support *D. brasiliensis* as a viable source of 9‐DOM, a promising hit for subsequent optimization in drug discovery.

## 4 Experimental Section

### 4.1 General Procedures

Silica gel (230–400 mesh) was used for column chromatography procedures while silica gel 60 PF_254_ was employed for analytical TLC separations. NMR spectra were recorded using a Varian INOVA spectrometer operating at 500 and 125 MHz for ^1^H for ^13^C nuclei, respectively, using CDCl_3_ as the solvent and TMS as the internal standard. HR‐ESIMS spectra were recorded using Bruker Daltonics MicroTOF QII spectrometer operating in positive electron spray ionization mode.

### 4.2 Plant Material

Branches of *D. brasiliensis* were collected in December 2021, in *Serra do Cipó* National Park, Minas Gerais, Brazil. The species was identified by the botanist Dr Guilherme M. Antar from Federal University of Espírito Santo, Brazil. The voucher specimen, registered as number 4105, was deposited at the herbarium of the University of São Paulo (SPF), Brazil.

### 4.3 Extraction and Isolation

As previously reported [5], fresh branches of *D. brasiliensis* were dried at 30°C and powdered to afford 316 g of plant material, which was extracted with hexane (10 × 500 mL) at room temperature. Combined extracts were concentrated under reduced pressure to afford 17 g of hexane extract. Part of this extract (16 g) was chromatographed over silica gel eluted with increasing amounts of EtOAc in hexane (9:1, 8:2, 7:3, 6:4, and 3:7) and pure EtOAc to afford five groups (A–E). Part of Group E (200 mg) was subjected to further fractionation using silica gel soaked with AgNO_3_ eluted with hexane:Et_2_O 7:3 and 1:1 to afford 22 mg of 9‐DOM.

*9‐DOM*: White amorphous solid. HR‐ESIMS: *m*/*z* 233.1557 [M+H]^+^ (calculated for C_15_H_21_O_2_^+^ 233.1541). ^1^H NMR (CDCl_3_, 500 MHz): *δ* 9.53 (d, *J* = 4.2 Hz, H‐11), 9.50 (s, H‐12), 7.14 (m, H‐7), 4.91 (br s, H‐13_eq_), 4.72 (br s, H‐13_ax_), 3.01 (br s, H‐9), 2.44 (m, H‐6), 2.11 (m, H‐5), 2.02 (m, H‐3), 1.91 (dt, *J* = 13.5 and 3.1 Hz, H‐1_eq_), 1.70 (m, H‐2_eq_), 1.62 (m, H‐1_ax_), 1.08 (d, *J* = 6.5 Hz, H‐14), 0.73 (s, H‐15). ^13^C NMR (CDCl_3_, 125 MHz): *δ* 201.2 (C‐11), 193.4 (C‐12), 153.0 (C‐7), 151.4 (C‐4), 138.0 (C‐8), 106.1 (C‐13), 58.4 (C‐9), 45.9 (C‐5), 39.5 (C‐1), 38.6 (C‐3), 38.3 (C‐10), 31.6 (C‐2), 27.1 (C‐6), 18.5 (C‐14), 13.6 (C‐15) (Supporting information).

### 4.4 In Silico ADME Analysis

In silico parameters (physicochemical descriptors, pharmacokinetic properties, and drug‐likeness) were evaluated using the SwissADME platform developed and maintained by the Swiss Institute of Bioinformatics, Lausanne, Switzerland (https://www.swissadme.ch/; accessed in 2025) [18]. The two‐dimensional structures of the analyzed compounds were drawn using the Marvin JS molecular editor (ChemAxon), based on Simplified Molecular Input Line Entry System (SMILES) representations obtained from PubChem, for subsequent properties predictions. The analysis also included screening for PAINS.

### 4.5 Determination of In Vitro Cytotoxicity

To evaluate the cytotoxicity of the compound and determine the optimal concentration for further experiments, the tetrazolium salt colorimetric (MTT) assay was conducted [50]. RAW246.7 cells were seeded at a density of 2 × 10^5^ cells/mL in 96‐well plates and incubated for 24 h in RPMI medium supplemented with 5% FBS at 37°C in a 5% CO_2_ atmosphere. Following incubation, the cells were treated for 24 h with the compound at concentrations of 200, 100, 50, 25, 12.5, 6.3, 3.1, 1.6, 0.8, 0.4, 0.2, and 0.1 µg/mL, as well as 50, 25, 12.5, 6.3, 3.1, and 1.6 ng/mL in medium containing 5% FBS. Positive control cells were treated with medium containing 5% FBS alone, while negative control cells were treated with 10% DMSO. To assess cell viability, the supernatant was removed, and 100 µL of MTT solution (0.5 mg/mL in RPMI) was added to each well and incubated for 4 h. The resulting formazan crystals were dissolved in 100 µL of isopropanol, and the optical density was measured at 570 nm using a microplate reader.

### 4.6 Animals and Ethics Procedures

Sixty‐four male C57BL/6 mice, 7–8 weeks (20–25 g body weight), were provided by Rede de Biotérios de Roedores from Universidade Federal de Uberlândia (REBIR/UFU). The animals were placed in isolators in groups of up to five mice. After the surgical implantation of the sponge discs, they were separated, keeping only one animal per isolator. Throughout the experiment, the animals had free access to food and water and were kept under controlled conditions (22°C, 60%–65% relative air humidity; 12 h light/dark cycle). The entire experimental protocol was approved by the Ethics Committee for the Use of Animals at the Federal University of Uberlândia (protocol number 23117.042533/2024‐32).

### 4.7 Preparation of Sponge Discs, Implantation, and Treatment

Polyether–polyurethane sponge discs (Vitafoam Ltd. Manchester, UK) with 5 mm thick × 8 mm diameter were aseptically implanted into a subcutaneous pouch. The sponge discs were soaked overnight in 70% v/v EtOH and sterilized by boiling in distilled water for 30 min. Before surgery all animals were anesthetized by intraperitoneal injection of a mixture of ketamine (100 mg/kg) and xylazine (10 mg/kg), the skin on the back was shaved and then wiped with 70% v/v EtOH. One sponge disc per animal was implanted through a 1 cm long dorsal mid‐line incision near the base of its tail. The incisions were closed with a silk braided non‐absorbable suture. The sesquiterpene 9‐DOM at 0.1, 1, or 10 µg in 10 µL of 0.5% DMSO was daily injected within the implants (intra‐implant), during nine days, starting immediately after sponge implantation. The control group received only 0.5% DMSO (vehicle) injections (10 µL). On the nineth day after surgery, animals were euthanized with an overdose of anesthetic (300 mg/kg of ketamine and 100 mg/kg of xylazine) and implants were surgically removed, weighed and processed for biochemical and histological analysis.

### 4.8 Biochemical Determination of Hemoglobin Content

Part of the implants were weighed and homogenized in 2 mL of Drabkin reagent (Labtest, Brazil). Next, samples were centrifuged at 12 000 × *g* for 40 min at 4°C. The supernatants were carefully collected and filtered through a 0.22 µm Millipore filter. A standard curve, of known hemoglobin concentrations, and samples were pipetted in duplicate into a 96‐well plate [51, 52]. Hemoglobin was quantified by reading the absorbance at 540 nm.

### 4.9 Quantifying Soluble Collagen Levels

After being weighed, part of each implant was homogenized in 1 mL of saline Triton X‐100 solution. The samples were then centrifuged at 6000 × *g* for 10 min at 4°C and 25 µL of the supernatant was transferred to another microtube, where 25 µL of *Sirius Red* reagent was also added. The samples were incubated at room temperature for 30 min. The collagen‐dye complex was precipitated by centrifugation at 10 000 *× g* for 10 min at 4°C. The supernatants were discarded and the pellet washed with 500 µL of EtOH (99% pure and methanol free). One milliliter of a NaOH solution (0.5 M) was added to the remaining pellet of collagen‐bound dye. After solubilization, samples were transferred to a 96‐wellplate, in duplicates, and read at 540 nm using a spectrophotometer [53]. The calibration curve was set up on the basis of a gelatin standard (Merck, USA).

### 4.10 Tissue Extraction and Determination of MPO and NAG Activity

After measuring the hemoglobin content, the previously stored samples was thawed and centrifuged at 15 300 × *g* for 15 min at 4°C. The sponge fragment was recovered and divided. Each part was then weighed and processed to measure MPO or NAG enzyme activity. To assess MPO activity, the implants were weighed and homogenized in sodium phosphate buffer (pH 6.0, 80 mM). Three hundred microliters of each sample were collected and added to 600 µL of 0.05 M Na_3_PO_4_ buffer (pH 5.4) containing 0.5% hexadecyltrimethylammonium bromide (HTAB). The samples were then ultrasonicated for 40 s and subjected to three freeze‐thaw cycles in liquid nitrogen. The samples were centrifuged (2700 × *g* for 10 min at 4°C) and MPO activity was evaluated from 200 µL of the supernatants by measuring the change in absorbance (optical density; OD) at 450 nm using tetramethylbenzidine (1.6 mM) and H_2_O_2_ (0.3 mM). The reaction was terminated by the addition of 50 µL of H_2_SO_4_ (4 M). The results are expressed as change in OD per gram of wet tissue [54, 55].

To access NAG activity, the samples were homogenized in NaCl solution (0.9% w/v) containing 0.1% v/v Triton X‐100 (Promega, Madison, WI, USA) and centrifuged (960 × *g* for 10 min at 4°C). One hundred microliters of the supernatant was collected and incubated for 1 h at 37°C with 100 µL of *p‐*nitrophenyl‐*N*‐acetyl‐β‐d‐glucosaminide (2.24 mM) (Sigma‐Aldrich, St. Louis, MO, USA), prepared in citrate/phosphate buffer (0.1 M citric acid, 0.1 M Na_2_HPO_4_; pH 4.5). At the end of this period, the reaction was stopped by adding 100 µL of 0.2 M glycine buffer (pH 10.6). Substrate hydrolysis was determined by measuring absorbance at 400 nm (nmol/mg wet tissue) [55].

### 4.11 Histological Processing and Analysis

After removal, the implants were fixed in Methacarn (60% v/v methanol, 30% v/v cheloroform, 10% v/v acetic acid) at 4°C for 3 h. After embedding in paraffin, 5‐µm thick slices were obtained in a rotating microtome (MICROM/HM‐315). For each animal, four histological sections were obtained. These were stained with H&E, toluidine blue or picrosirius red, for light microscopy studies. To quantify the blood vessels, the sections were stained with H&E and processed for light microscopy studies. A countable vessel was defined as a structure with a lumen, whether or not it contained red blood cells. Images of 20 continuous fields from each implant were evaluated. Mast cell density was determined by counting these cells in sections stained with toluidine (also 20 sequential fields from each implant). For these two analyses, the images were digitized using a Leica ICC50 micro camera (40× objective; 400× final magnification). The sections stained with picrosirius red were captured and digitized with a Nikon TS 100 microscope (20× objective; final magnification 200×) coupled to an Opticam micro‐camera system. Images were captured with and without the use of a polarizing filter. Under polarized light it is possible to distinguish thinner collagen fibers (green) from thicker fibers (yellow/orange). Twenty random areas of each slide, one slide per animal, were analyzed. The average obtained after the quantification of these areas (total value of all areas/20) was used for statistical analysis. Thus, it was possible to evaluate both the deposition, organization and maturation degree of collagen fibers. The images were analyzed with the aid of ImageJ software (National Institutes of Health—NIH).

### 4.12 Statistical Analysis

The data were presented as mean ± standard error of the mean. Comparisons between groups were made using the single one‐way analysis of variance (ANOVA), followed by the Bonferroni as a posttest. Differences between means were considered significant when *p* < 0.05. The statistical analysis and the construction of graphics were performed using the GraphPad Prism program, ver. 7.0.

## Author Contribution

**Bruno Antonio Ferreira**: conceptualization, formal analysis, investigation, methodology, project administration, visualization, writing – original draft. **Isabella Silva Cassimiro**: conceptualization, formal analysis, investigation, methodology, project administration, visualization, writing – original draft. **Francyelle Borges Rosa de Moura**: formal analysis, investigation, methodology. **Tais de Campos Lima**: formal analysis, investigation, methodology. **Danielle Reis Napolitano**: formal analysis, investigation, methodology, resources. **Eric Umehara**: formal analysis, investigation, methodology. **João Henrique Ghilardi Lago**: conceptualization, formal analysis, funding acquisition, project administration, resources, supervision, validation, writing – review and editing. **Fernanda de Assis Araújo**: conceptualization, formal analysis, funding acquisition, project administration, resources, supervision, validation, writing – review and editing.

## Conflicts of Interest

The authors declare no conflicts of interest.

## Supporting information




**Supporting File 1**: cbdv70830‐sup‐0001‐SuppMat.docx

## Data Availability

The data that support the findings of this study are available from the corresponding author upon reasonable request.
