# Supplementary material for: 9‐Deoxymuzigadial, a Sesquiterpene Isolated From Drimys brasiliensis (Winteraceae), Displays Reduced Cytotoxicity In Vitro and Modulates Leukocyte Activity and Fibrogenesis In Vivo
Source: Chem Biodivers. 2026 Jan 8;23(1):e03329. doi: 10.1002/cbdv.202503329 (PMC12781153; doi:10.1002/cbdv.202503329)
Supplement: Supplementary file 1 — Supporting File 1: cbdv70830‐sup‐0001‐SuppMat.docx [file CBDV-23-e03329-s001.docx]

**9-Deoxymuzigadial, a Sesquiterpene Isolated from *Drimys brasiliensis* (Winteraceae) Displays Reduced Cytotoxicity *In Vitro* and Modulates Leukocyte Activity and Fibrogenesis *In Vivo***

Bruno Antonio Ferreira^a^, Isabella Silva Cassimiro^b^, Francyelle Borges Rosa de Moura^c^,

Tais de Campos Lima^b^, Danielle Reis Napolitano^b^, Eric Umehara^a^,

João Henrique Ghilardi Lago^a^*, Fernanda de Assis Araújo^d^*

^a^ Center for Natural and Human Sciences, Federal University of ABC, São Paulo 09210-170, Brazil

^b^ Institute of Biomedical Sciences, Federal University of Uberlândia, Uberlândia, 38408-100, Brazil

^c^ Institute of Biotechnology, Federal University of Catalão, Catalão, 75704-020, Brazil

^d^ Federal University de São João Del-Rei, Divinópolis, Brazil.

* Corresponding authors: [joao.lago@ufabc.edu.br](mailto:joao.lago@ufabc.edu.br) and [fearaujo@ufsj.edu.br](mailto:fearaujo@ufsj.edu.br)

**
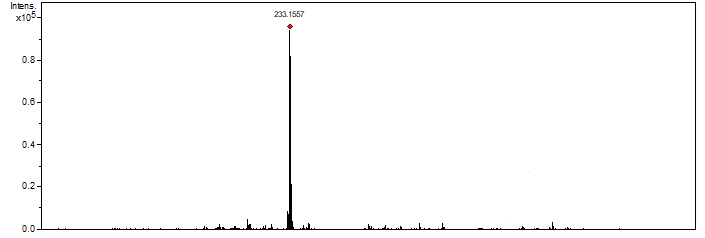
**

**Figure S1**: ESI-HRMS of 9-deoxymuzigadial

**Figure S2.** ^1^H NMR spectrum of 9-deoxymuzigadial (δ, CDCl_3_, 500 MHz)

**Figure S3.** ^13^C NMR spectrum of 9-deoxymuzigadial (δ, CDCl_3_, 125 MHz)


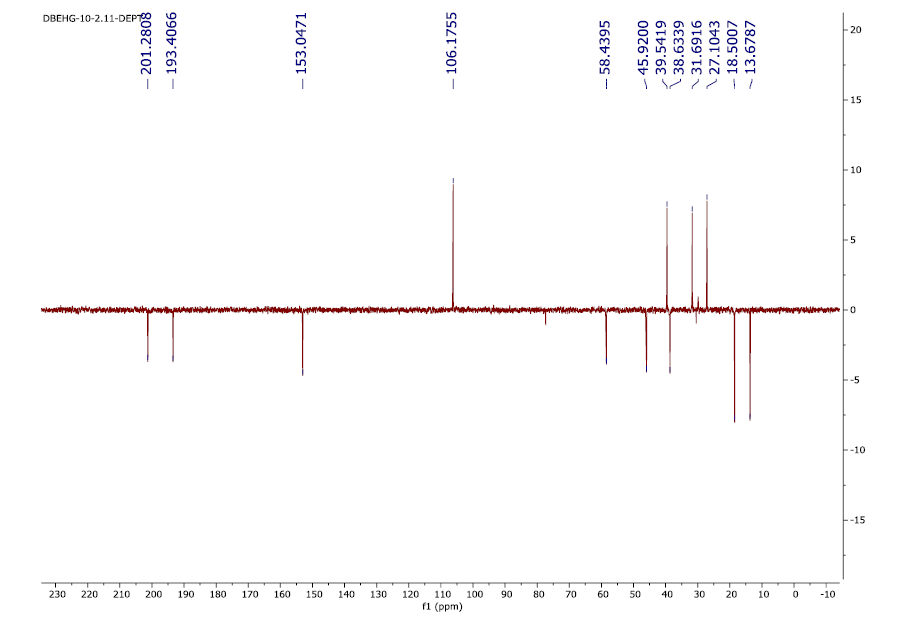


**Figure S4.** DEPT NMR spectrum of 9-deoxymuzigadial (δ, CDCl_3_, 125 MHz)
